# Hollow-core antiresonant THz waveguides based on polymer- and metal-coated sapphire tube

**DOI:** 10.1038/s41598-026-50305-8

**Published:** 2026-04-27

**Authors:** Gleb M. Katyba, Anna S. Kucheryavenko, Alexander N. Golikov, Anna N. Rossolenko, Vladimir N. Shilov, Kirill B. Dolganov, Maria G. Burdanova, Igor E. Spektor, Vladimir N. Kurlov, Kirill I. Zaytsev

**Affiliations:** 1 Osipyan Institute of Solid State Physics of the Russian Academy of Sciences, 142432 Chernogolovka, Russia; 2 Prokhorov General Physics Institute of the Russian Academy of Sciences, 119991 Moscow, Russia; 3 Moscow Center for Advanced Studies, 123592 Moscow, Russia

**Keywords:** Materials science, Optics and photonics, Physics

## Abstract

While the existing terahertz (THz) fiber optics components suffer from high loss and dispersion, low technological reliability, poor environmental resistance and radiation strength, as well as large cross-section, THz applications in different fields still require hardware for the sensing and exposure of hard-to-access objects. To mitigate this difficulty, we develop the two variants of hollow-core THz waveguides, those exploit the antiresonant reflecting optical waveguiding (ARROW) mechanism and use (as a key element) a few-millimeter-diameter sapphire tube produced by the edge-defined film-fed growth (EFG) technique. In the all-dielectric arrangement, the outer surface of this tube is coated by a sub-millimeter-thick polytetrafluoroethylene (PTFE) film, while in the metal-coated one—by a sub-micrometer-thick reflecting copper layer. These coatings increase the guiding efficiency and underlie different performance of the two geometries. Both waveguides are studied numerically and experimentally in the 0.56–0.7 THz frequency range. The observed discrepancies between the theoretical and measured propagation loss are attributed to fluctuation of the cross-section geometry over the waveguide length. In narrow frequency bands, the metal-coated waveguide offers the propagation loss as small as 5.0 dB/m, which is significantly lower than that of the all-dielectric one. Furthermore, the outer metal coating completely prevents mode leakage, whereas in an all-dielectric waveguide, some of the evanescent field extends into the surrounding space and still can be de-coupled. Our findings highlight that the ARROW sapphire THz waveguides provide a reasonable compromise between the guiding efficiency and the cross-section dimensions, thus, forming a favorable platform for the THz sensing and exposure.

## Introduction

Nowadays, THz technologies are vigorously explored^1–4^ thanks to their great potential in non-destructive testing^5,6^, quality control^7,8^, 6G communications^9,10^, medical diagnosis and therapy^11–15^, and others. However, translation of THz technology into all these practical fields is hampered by the lack of efficient THz fiber optics (rigid waveguides, flexible fibers, optical fiber bundles, endoscopes, etc.), which is mostly due to the limit set of available efficient THz optical materials and related fabrication strategies^16–18^. For example, most THz biomedical applications are impossible without the THz endoscopic systems capable of the THz-wave delivery to hard-to-access tissues and internal organs spaced at the distance of a few tens of centimeters from the THz equipment^19–22^. In fact, there is still no feasible way to produce THz fibers, which simultaneously guide radiation over a considerable distance (with low propagation loss and dispersions), have a small cross section, offer chemical and radiation robustness, mechanical strength, technological reliability, cost-efficiency, and (preferably) flexibility  ^17^. The existing material platforms of THz fiber optics presume an interplay between the aforementioned list of properties  ^4^.

Among the most promising methods of THz fiber fabrication is polymer extrusion, which enables the production of long waveguides with fairly complex cross sections. Polymers with low THz absorption, such as TOPAS ^23,24^ and ZEONEX ^25^, are commonly used as a material platform for the polymer-extruded THz ARROW waveguides. Recently, the propagation losses as low as 3 dB/m (by field) at 0.9 THz and 15 dB/m at 0.4 THz were reported for such waveguides ^23^. The record propagation loss of $\le 1~$dB/m at 0.6 THz was reported for another polymer-extruded THz ARROW waveguide ^26^, but its outer diameter of 16.5 mm seems to be too large for a variety of THz applications, such as endoscopy. Waveguides of this type are biocompatible. However, they can withstand temperatures only up to $150~^\circ$C, which also imposes some constraints on their applicability in many fields.

Another approach to waveguide/fiber fabrication is 3D printing of polymer structures ^27^. In recent years several techniques of waveguide fabrication have been developed, including high-resolution photopolymerization ^28^,material jetting ^29^ and powder bed fusion ^30^. In particular, the stereolithography-based 3D printing technique enables high-precision printing (with a resolution of $\approx 10~\mu$m) ^28^ of waveguide with an average transmission loss of 48 dB/m in the range of 0.2 to 1 THz and a minimum transmission loss of $\approx 5.2$ dB/m at 0.922 THz. Powder bed fusion methods are suitable for the production of THz filters based on the rectangular metal waveguides  ^31^. For relatively low THz frequencies ($\sim 0.1$ THz), such additive manufacturing process provides comparable device performance, but for higher frequencies, further improvements are needed. Material jetting forms an alternative approach for the fabrication of polymer THz fibers. For example, in paper ^32^, flexible fibers with the length of $\le 5~$m were reported. They exhibit the propagation loss of 10 dB/m in the $0.12-0.15$ THz range, along with low bending losses and compact (6.8 mm) outer diameter. These fibers represent a promising platform for the short-range 6G communications at the frequencies $<0.15$ THz. Additionally, they show potential in THz biophotonics.

Recently, as a favorable materials platform of the THz optics, the EFG-grown sapphire waveguides were considered by our team^4,16,33^. The EFG method facilitates the production of shaped crystals with a complex cross-section and a $\simeq 1$-m-length, sufficient for the THz^19,22^. The main advantages of sapphire shaped crystal, as a material platform of the THz waveguide optics, are the high transparency in the THz range, biocompatibility and inertness to the outer conditions (including the high temperatures) ^4,16^).In fact, the operating temperature of sapphire instruments might reach $1600~^{\circ }$C, which broadens their applicability in many fields. Despite the step-index sapphire fibers are not suitable for the THz waveguidence over the $\simeq 10$-cm-long distances due to the guided-mode confinement in the core material, leading to attenuation and dispersion ^34^ and, thus, are poorly suitable for endoscopy, the as-grown hollow-core sapphire shaped crystals with a multichannel (or multilayer) cladding and a guided-mode confinement in a hollow core can realize either the photonic crystal/Bragg^35^ or ARROW^36^ waveguiding mechanisms with low dispersion and propagation loss in a broad spectral range. Another notable advantage of such waveguides relies on the unique properties of sapphire: high refractive index and overall low absorption at THz frequencies, chemical inertness, radiation and mechanical strength, biocompatibility, etc. While the photonic crystall waveguidance require very large dimensions of the cladding (i.e., a few lattice periods – $\ge 10 \lambda$) and, thus, appear to be less suitable for endoscopy, ARROW principle slightly reduces the dimension of the cladding (to the level of $\simeq \lambda$), making them suitable for endoscopy. It enables low propagation loss when the antiresonance condition is satisfied across multiple frequencies^37–39^. Also, ARROW waveguides offer considerable potential for the optimization of guiding properties via the judicious design of cross-section^40,41^, but also demand for the highly-accurate ($\sim 0.1 \lambda$) maintenance of the cross-section geometry over the waveguide length^41^. However, the discussed potential of optimization and demands on accuracy still have not been studied for the sapphire ARROW THz waveguides.

To address this challenge and better analyze capabilities of the EFG-grown sapphire ARROW THz waveguides in endoscopy, in this paper, we develop the hollow-core ARROW THz waveguides featuring the two distinct geometries. Both use a few-millimeter-diameter sapphire tube, as a key element. They are designed for the 0.56–0.7 THz range to compete with other THz ARROW waveguides – i.e., those based on the polyether-ether-ketone tube with an outer copper coating^42,43^ and other all-dielectric arrangements^44–47^. While the first waveguide (i.e., all-dielectric) uses the sub-mm-thick PTFE coating of a tube (as reported in Refs.^19,36^), the second (i.e., hybrid or metal-coated) one is reported for the first time and uses the sub-$\mu$m-thick reflecting copper coating. Distinct coatings ensure the guiding efficiency, eliminate the mode leakage, and underlie different performance of these waveguides. The waveguides are studied numerically and experimentally, while the observed discrepancies between the theory and measurements are attributed to deviations of the waveguide cross-section from the desired one. For the metal-coated arrangement, the minimal propagation loss is as small as $\alpha = 1.2$ dB/m (by power) at the frequency of $\nu = 0.597$ THz, which overall agrees with the numerically predicted value of 0.9 dB/m. In turn, for the all-dielectric waveguide, the measured loss is seven times higher than that for the metal-coated one. Our findings highlight prospects of the sapphire ARROW THz waveguides: their high optical performance, along with their technological reliability, makes them a promising platform for the THz endoscopy.

## Design

**Figure 1:**
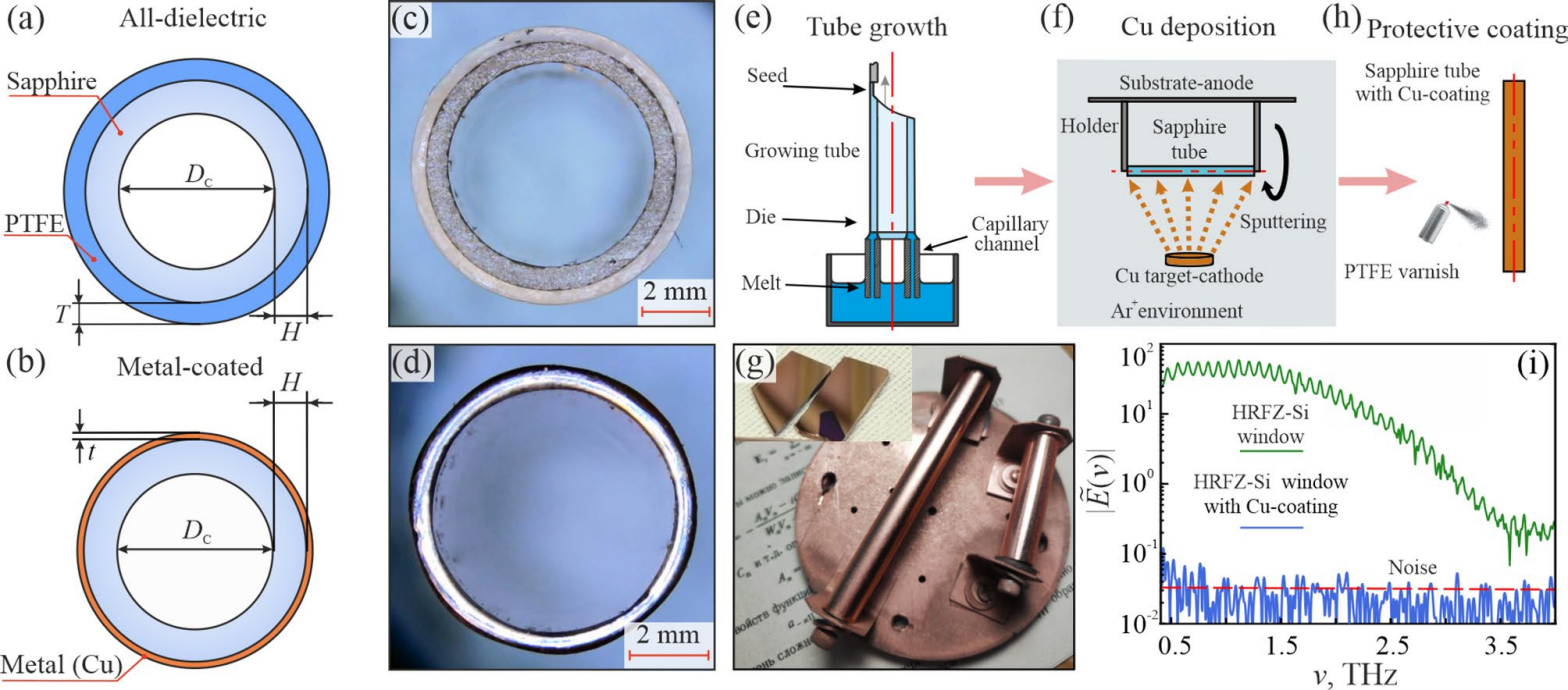
Design and fabrication of the ARROW THz waveguides. (**a**–**d**) Schemes and photos of the cross-sections of the all-dielectric and metal-coated waveguides. (**e**) Scheme of the EFG growth of the sapphire tube. (**f**) Scheme of the 600-nm-thick copper coating sputtering onto the outer surface of the rotating sapphire tube. (**g**) Photo of the metal-coated sapphire tubes in the rotating holder after the sputtering process, where insert shows the reference HRFZ-Si windows, used for the sputtering and spectroscopy of an equal metal coating. (**h**) Scheme of the protective coating formation on the metal-coated waveguide. (**i**) Transmission (by field) of the empty HRFZ-Si window and that with the 600-nm-thick copper coating.

Both the all-dielectric and metal-coated waveguides use an equal sapphire tube with the desired inner diameter of $D_\textrm{C} = 6.15$ mm, the wall thickness of $H = 0.73~$mm, and the sapphire *c*-axis directed along the waveguide optical axis; see Figs. 1a,b, respectively. In the all-dielectric waveguide, PTFE shrink film with the thickness of $T = 0.48~$mm covers the sapphire tube, works as the second antiresonance layer, and prevents the guided-mode de-coupling into the surrounding space (owing to the interactions between its evanescent field and external obstacles).

For the ARROW mechanism, minima in the propagation loss $\alpha \left( \nu \right)$ appear under the antiresonant conditions at certain frequencies $\nu$, at which the destructive interference of waves within the cladding causes their back-reflection into a core. The thickness of *q*-th cladding layer in such a waveguide can be approximately calculated as^41^

1 $$\begin{aligned} t_\textrm{qp} \approx \frac{ p \pi }{ 2 \sqrt{ \frac{ 4 j_\mathrm {g\mu }^2 }{ D_\textrm{C}^2 } + \left( \frac{ 2 \pi }{ \lambda } \right) ^2 \left( n_\textrm{q}^2 - 1 \right) } }, \end{aligned}$$

where $j_\mathrm {\mathrm {g\mu }}$ is the $\mu$-th zero of the 1-st-order Bessel function $J_\textrm{g}$, $\lambda$ is the free-space wavelength, $n_\textrm{q}$ is the refractive indexes of *q*-th cladding layer (in our case, they are formed by sapphire^48^ and PTFE^49^ materials), *p* is a positive even integer when $n_\mathrm {q-1}^2<n_\textrm{q}^2<n_\mathrm {q+1}^2$ and , otherwise the odd values of *p* lead to antiresonance and even ones to resonance.

In our study, (1) is used to design the waveguides with minimal propagation loss near $\lambda = 600$ $\mu$m, taking into account the technological limitations posed by the EFG technique. Then, in more-accurate numerical analysis, we account for the optical anisotropy of sapphire, material absorption and dispersion, as well as possible geometric deviations in the cross-section geometry. Namely, such factors, as fluctuations in the thickness of sapphire tube and PTFE film over the length, those can lead to considerable degradation of the waveguide optical properties, are addressed.

## Fabrication

The approach for the sapphire tubes’ growth is based on the EFG technique  ^50^. It involves the crystal growth from a thin melt film formed on the top of a wettable molybdenum die. The melt rises from the crucible through the $0.1-0.3$-mm-width capillary channel to the top surface of the die thanks to capillary forces. In this way, a thin melt film spreads over this surface of the die, being constrained by the die edges (Fig. 1e). Geometry of the as-grown crystal cross-section is mainly determined by the die geometry, but it can be varied in narrow limits defined by the stability region of the melt meniscus pinned at the free edge of the die. A comprehensive description of the EFG process (as well as of its modifications) in the context of sapphire shaped crystal growth can be found in Refs. ^16,33,51,52^. This method enables the production of both the large-diameter sapphire tubes (up to 85 mm) ^53^ and thin-walled capillaries ^54,55^.

To manufacture the considered sapphire tubes (those serve as key components of THz ARROW waveguides) the commercially available growth setup NIKA (EZAN, Chernogolovka, Russia) was used. It includes a growth chamber maintained under a high-purity Ar atmosphere at a pressure of $1.1-1.3$ atm, and a molybdenum crucible heated by a 22 kHz induction-heated graphite susceptor. Crushed Verneuil boules were used as the charge material for sapphire tube growth. C-axis ($<0001>$) oriented seeds were employed to initiate crystallization of sapphire tubes. The pulling rates were varied in the 60–70 mm/h range. When the seed crystal touches the upper edge of the molybdenum die, a $0.2-0.3$-mm-height melt meniscus is formed between them. The seed is then pulled upward into a cooler region, where a tubular crystal begins to form. A weight sensor-based automation of the growth process ^52,53^ makes it possible to produce a crystal with a very smooth as-grown surface and minimizes variations of the tube thickness along its length.

However, since the melt zone has a viscosity close to that of water, deviations from the ideal tube shape can occur due to vibrations and irregular motions in the growth system. The dimensional inaccuracies of the die also directly impact the resultant tube geometry. For the THz applications, sapphire tubes with the lengths of tens of centimeters and the wall thickness of $\ge 300~\mu$m can be produced with the acceptable sub-wavelength accuracy of $0.1~\lambda$ (here, $\lambda \sim 600~\mu$m) by combining the EFG method with mechanical post-processing. Sufficiently long sapphire tubes with very thin walls are rather difficult to post-process due to their relatively high fragility. Conversely, increasing the wall thickness leads to higher outer diameter of the waveguides. Therefore, in this study, the particular value of wall thickness *H* near 0.7 mm was selected as a compromise that ensures both the technological reliability and the high optical performance of the resultant waveguides.

The technological advantages of the EFG-grown sapphire shaped crystals, along with a unique combination of their physical properties, make them a favorable THz optical material platform, as compared to other crystalline THz optical materials. For example, it is not possible to produce high-quality and low-loss shaped silicon crystals (for example, the silicon tubes) by the EFG technique, or others. Thus, commonly, only a small-scale THz optical components are made of silicon by mechanical shaping (machining) of a bulk crystal obtained using the Float Zone method ^56^.

To form the all-dielectric waveguides, the two fragments of the sapphire tube, with the lengths of 50 and 100 mm and polished surfaces (roughness level is about $\le 0.01 \mu$m), are covered by the polymer cladding via the PTFE-tube shrinkage, as detailed in Refs.^19,22,36^. Such a coating also has fluctuations in its thickness due to those in the initial thickness of PTFE tube and other factors. In turn, to form the metal-coated arrangement, the copper coating is deposited onto the sapphire tube using the in-house vacuum sputtering setup (ISSP RAS); see Fig. 1f. For this, the two sapphire tubes, with the lengths of 35 and 65 mm, are fixed in a judiciously-designed holder, which allows for their rotation during the sputtering process and prevents the Cu deposition onto the inner tube walls (panel (h)). The basis of this holder is the metallic constrictive rod with the internal M4 thread and the diameter slightly smaller than the inner diameter of sapphire tube. The sapphire tube was fixed in the holder using this rod and the pair of screws, while the PTFE washers ensure a tight fit with the edges of sapphire tubes. The copper coating is deposited at a few discrete rotation angles, which ensures the homogeneous coating thickness of $t=600$ nm (it is much larger than the skin depth $\Delta \approx 90~$nm in bulk copper). This parameter is directly measured using the atomic-force microscope OMICRON VT AFM XA (ISSP RAS) of the copper-coated planar HRFZ-Si window from the same sputtering run. From panel (i), one notices that such a window is optically opaque in the THz range, as justified experimentally using the in-house transmission-mode THz pulsed spectrometer (GPI RAS). Finally, a PTFE anti-friction varnish with an acrylic binder is then applied to the outer surface of the metal-coated waveguide (panel (g)). After the PTFE varnish deposition on the outer surface, the waveguides were removed from the holder and additionally washed by the ethanol.

In Fig. 1c,d, we show photos of the cross-sections of thus fabricated all-dielectric and metal-coated waveguides, respectively. The resultant geometrical parameters of the cross-sections are estimated as $D_\textrm{C} = 6.16 \pm 0.03$ mm, $H = 0.72 \pm 0.12$ mm, and $T = 0.47 \pm 0.15$ mm. These geometrical definitions form the basis for our numerical analysis.

## Numerical analysis

**Figure 2:**
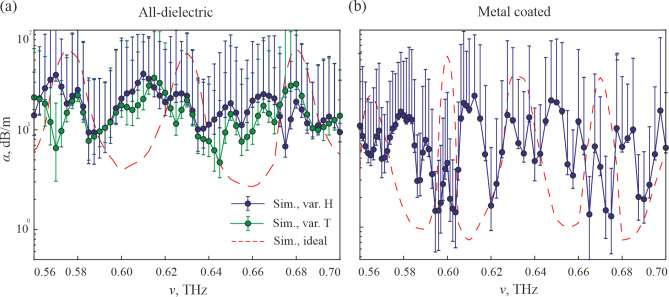
Numerical analysis of the propagation loss in the ARROW THz waveguides. (**a**) Propagation loss $\alpha \left( \nu \right)$ (by power) for all-dielectric waveguide. The red dotted line represents the ideal waveguide with the desired geometry. The blue and green curves illustrate the impact of variation in the thicknesses of sapphire and PTFE layers, respectively, while the solid circles represent the mean loss values for different waveguides geometries and error-bars represent the band between minimal and maximum loss values. (**b**) Equal data set for the metal-coated waveguide, where only the variations in sapphire layer thickness are considered.

The propagation loss $\alpha \left( \nu \right)$ (by power) is numerically calculated for the fundamental core-guided mode of our waveguides using the finite-difference eigenmode method^57^ within the ANSYS mode software ^58^. For this, the optical properties of sapphire is taken from Ref.^48^, the commercial PTFE shrink tube is modeled as an annular polymer layer with a thickness *T* and optical properties from Ref.  ^49^, while the metal coating is considered as the semi-infinite Cu coating (its complex dielectric permittivity is taken from  ^59^). In our analysis, we consider different thicknesses of sapphire and PTFE layers.

In Fig. 2a,b, thus calculated $\alpha \left( \nu \right)$-curves are shown in the $\nu = 0.56$–0.70 THz range for the all-dielectric and metal-coated waveguides, respectively (in a logarithmic scale). The red dotted line represents the loss of an ideal waveguide with the desired dimensions. The all-dielectric waveguide has the minimal loss of 2.7 dB/m (by power) at 0.66 THz, as compared to the more-efficient metal-coated geometry offering 0.7 dB/m at 0.62 THz. The resonance peaks of high loss are observed for both waveguides upon the radiation coupling to the cladding modes. However, for the all-dielectric arrangement, some evanescent field still extends to the outer environment even on the anti-resonant conditions, causing a additional losses, and leading to the possible field de-coupling from the waveguide as well (upon a contact with external obstacles.) In the opposite case, the metal coating totally confines the field inside the waveguide, thus, the calculated $\alpha \left( \nu \right)$ has the more lower values in the anti-resonance band.

Sensitivity of the fundamental mode propagation loss to imperfections of the waveguide cross-section (i.e., fluctuations of its parameters over the length) is evaluated by performing a series of numerical calculations, as shown in Fig. 2.

For the all-dielectric waveguide, first, while maintaining the desired PTFE-coating thickness $T = 0.47$ mm, that of the sapphire tube is varied (with a uniform step) in the range of $H = 0.72 \pm 0.12$ mm, with a corresponding $\alpha _i \left( \nu \right)$-curve calculated at each *i*-th step. The average loss $\alpha (\nu )$ is then calculated for the fundamental mode of the imperfect waveguide as

2 $$\begin{aligned} \alpha \left( \nu \right) = \frac{ 1 }{ N } \sum _{i=1}^N \alpha _i \left( \nu \right) \end{aligned}$$

(here, $N = 5$ is the number of the calculated waveguide geometries), while the resultant propagation loss is shown in Fig. 2a in the form of the blue-colored mean curve and error bars. Here, the error bars represent the interval between minimal and maximal loss values observed in the independent simulations (but not the simple standard deviation). The minimal loss is observed at 0.59 THz, and it varies in the range of [5.2, 30.0] dB/m. Second, the green-colored curve and error bars are calculated (in the same manner) to highlight the evolution of loss with changes in the PTFE-coating thickness in the $T=0.47\pm 0.15$ mm range, while the sapphire tube thickness is maintained at $H = 0.72$ mm. Second, the green-colored curve and error bars are calculated (in the same manner) to highlight evolution of loss with changes in the the PTFE-coating thickness in the $T = 0.47 \pm 0.15$ mm range, while the sapphire tube thickness is maintained at $H = 0.72$ mm. After averaging of simulated curves, the minimal loss is found at 0.645 THz, with the variation interval of [3.3, 15.0] dB/m and the mean value of 4.7 dB/m. Evidently, such variations of *H* and *T* broaden and shift the resonant loss peaks in the $\alpha \left( \nu \right)$-curves, leading to an increase in the average loss across in the entire spectral range. and significant loss $\alpha \left( \nu \right)$ variations.

For the metal-coated waveguide, similar numerical analysis is performed considering the thickness of copper coating larger than the skin depth and varying only the sapphire tube thickness ($H = 0.72 \pm 0.12$ mm); see Fig. 2b. There is no noticeable de-coupling of THz radiation from the waveguide into the surrounding environment. Thus, the loss is lower that of an all-dielectric waveguide even under the anti-resonant conditions. For example, at 0.6 THz, the loss in the interval of 0.5, 3.8 dB/m with the mean value of 1.4 dB/m.

## Experiment

The two waveguides are studied experimentally. Namely, the propagation loss of fundamental guided mode is measured (in a double-pass scheme) by the in-house backward-wave oscillator (BWO) spectrometer, while the mode intensity distribution in the waveguide core is then visualized by the continuous-wave scanning-aperture near-field THz imaging.

**Figure 3:**
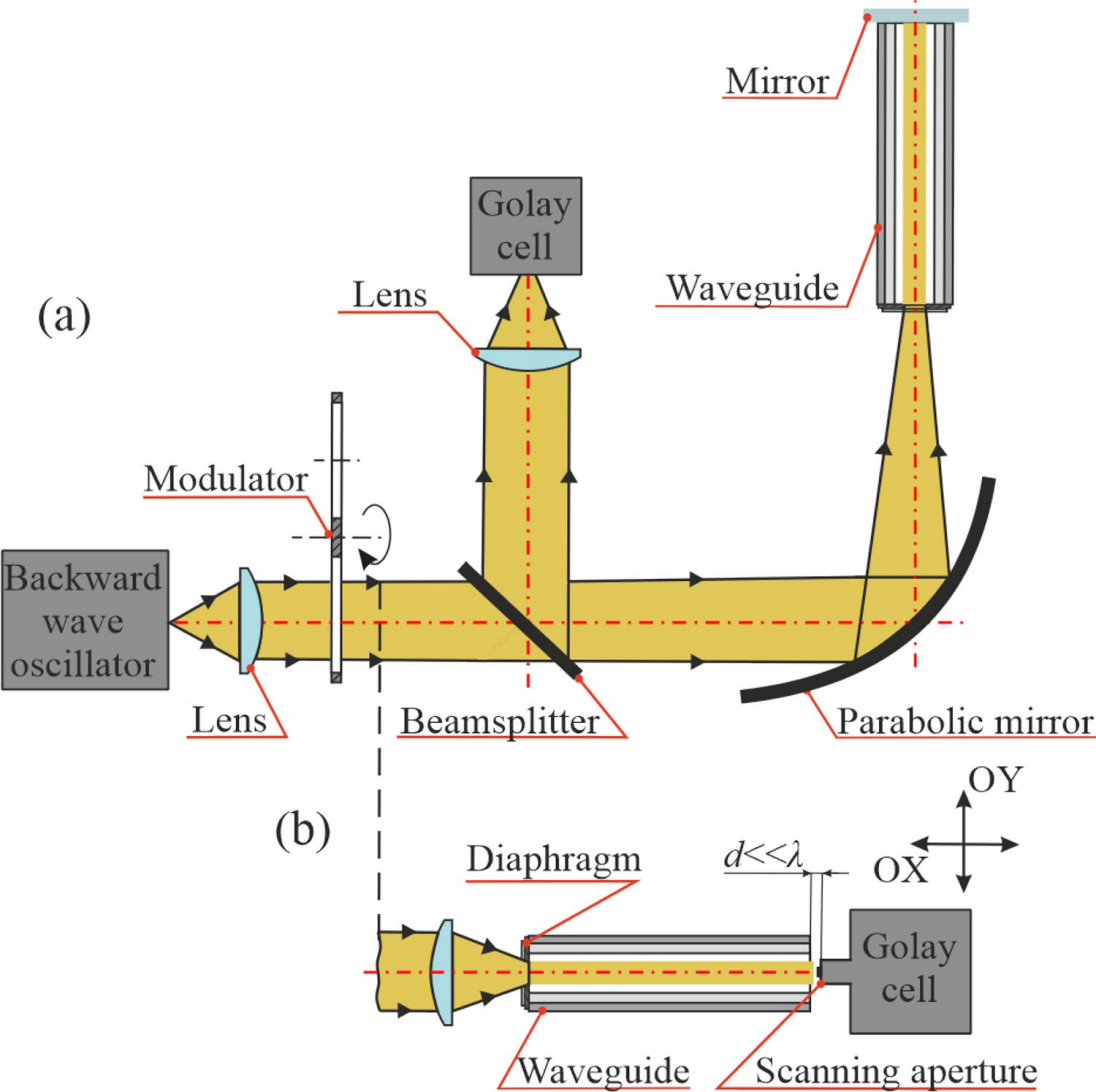
Experimental setup for the characterization of the ARROW THz waveguides. (**a**) Continuous-wave BWO spectroscopy of the propagation loss $\alpha (\nu )$ in the double-pass geometry. (**b**) Near-field imaging of the guided mode intensity distribution over the waveguide core using the scanning sub-wavelength aperture.

### Setups

From Fig. 3a, one notices that the THz-wave transmission through the waveguides of different lengths is studied by the in-house continuous-wave spectrometer that uses a back-ward wave oscillator^60^ and a Golay cell^61^, as an emitter and a detector of THz waves. The emitter has the output frequency tunable in the range of $\nu = 0.55$–0.70 THz, the linewidth of $\sim 10^{-4}$–$10^{-5} \nu$, and the average power of $\sim 10^{-3}$ W. The emitted THz beam is collimated by the plano-convex lens (made of the high density polyethylene – HDPE), passes through the 22 Hz mechanical chopper, and is focused onto the input waveguide edge by the off-axes parabolic mirror with the focal length of 100 mm and the diameter of 80 mm. The linearly-polarized THz beam with almost-Gaussian profile radiates the 4-mm-diameter metal aperture at the input waveguide end to excite the fundamental core-guided mode and prevent excitation of the high-order ones. The waveguide is fixed in the holder equipped by the 3-axis manual translation stage and rotating platform (not shown in the picture, for simplicity). The THz radiation propagates through the waveguide, is reflected by a flat gold mirror at the output waveguide end, and moves in the opposite direction. Thus, it passes the waveguide twice aimed at increasing the length of guided mode - waveguide interaction, obtain a more significant difference between the measured signals, and, thus, improving the sensitivity of measurements. Then, the THz wave left the waveguide, is collimated by the off-axis parabolic mirror, reflected by the beamsplitter, and then focused (by another HDPE lens) onto the Golay cell. By using the lock-in detection and by tuning the output frequency of our emitter, the frequency-dependent THz-beam power is detected for the further analysis of the waveguide optical performance.

For both the all-dielectric and metal-coated waveguides, the propagation loss $\alpha \left( \nu \right)$ is calculated from the measured THz power spectra of the two waveguide fragments with the distinct lengths, $l_{\textrm{1}}$ and $l_{\textrm{2}}$ (both on the order of tens of centimeters; see “Fabrication”)

3 $$\begin{aligned} \alpha \left( \nu \right) = - \frac{ 10 }{ 2 \left( l_{\textrm{2}}- l_{\textrm{1}} \right) } \lg \left( \frac{ R_2 \left( \nu \right) - R_\textrm{N} \left( \nu \right) }{ R_1 \left( \nu \right) - R_\textrm{N} \left( \nu \right) } \right) , \end{aligned}$$

where $R_1 \left( \nu \right)$ and $R_2 \left( \nu \right)$ are the measured power spectra for the waveguide lengths $l_{\textrm{1}}$ and $l_{\textrm{2}}$, and $R_\textrm{N} \left( \nu \right)$ is the (parasitic) THz reflection measured in the absence of a gold mirror at the output waveguide end. Particularly, $R_\textrm{N} \left( \nu \right)$ accounts for such effect as the THz-wave back-scattering from the input diaphragm or other element of the optical path. In (3), by normalizing the THz power spectral corresponding to the two distinct lengths, we exclude from our consideration the coupling and de-coupling loss, as well as all other factors those are equal for the two waveguide fragments. Five independent measurements for each waveguide type and length are performed to quantify both the average loss $\alpha \left( \nu \right)$ and the measurement errors defined by the standard deviation ($2\sigma$).

As shown in Fig. 3b, to visualize the guided-mode intensity in the waveguide cross-section, the in-house scanning-aperture near-field THz imaging system is applied, which uses the same BWO and Golay cell (as an emitter and a detector, respectively) and which is quite similar to the setups from Refs.^19,48,62^. For this, the waveguide is excited by the THz beam (in the same way as used in the spectroscopic measurements), the beam passes the waveguide once, and the 2D pixel-by-pixel lateral scanning is performed in the near-field zone – i.e., at the distance $d<< \lambda$ from the output waveguide end. The scanning is carried out by a Golay cell equipped with the 500-$\mu$m-diameter aperture and mounted on the 2D motorized translation stage with the scanning step of 250 $\mu$m and the positioning accuracy of $< 2$ $\mu$m. This helps us to visualize (in an identical manner) the modal intensity in the cross-sections of both waveguides.

### Comparison with numerical predictions

**Figure 4:**
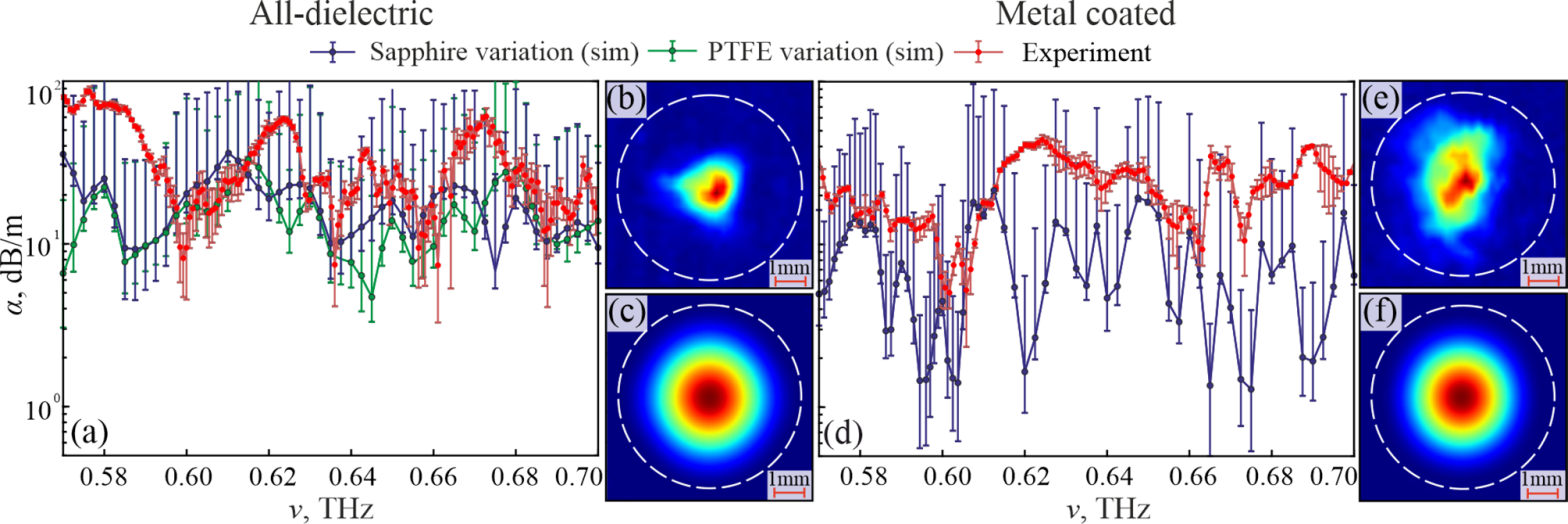
Experimental characterization of the ARROW THz waveguides. (**a**) Measured propagation loss $\alpha \left( \nu \right)$ (by power, in red) of the all-dielectric waveguide, as compared to the numerical predictions those account for the fabrication imperfections – variations in the thicknesses of sapphire (in blue) and PTFE (in green) layers. The error bars of experimental $\alpha \left( \nu \right)$-curve define the $2\sigma$ confidence intervals. (**b**,**c**) Experimental and numerical data on the guided-mode intensity distribution $I \left( \textbf{r} \right)$ over the waveguide core at 0.6 THz. The white dashed line defines the waveguide hollow core. (**d**–**f**) Equal data set for the metal-coated waveguide, where in (**d**), only the variations of sapphire layer thickness are considered.

In Fig. 4, the experimental propagation loss (Eq. (3)) shown in the 0.58–0.68 THz range and mode intensities at 0.6 THz are compared with the numerical predictions for the two waveguide geometries.

From Fig. 4a, one notices that the all-dielectric waveguide operates in the ARROW regime with oscillatory propagation loss $\alpha \left( \nu \right)$ and the minimal loss of $8.2 \pm 3.5$ dB/m (by power) at $\nu = 0.6$ THz. The measured losses are overall higher than the numerically-predicted values (particularly, near the resonant peaks), which can be attributed to such factors as the unaccounted variations in the waveguide shape, the residual excitation of higher-order guided modes, or the guided-mode leakage into the surrounding environment (due to the evanescent field – waveguide holder interactions), and others. Panels (b) and (c) show the experimental and numerical distributions, respectively, of the guided-mode intensity $I\left( \textbf{r} \right) \propto \left| \textbf{E} \right| ^{2}$ at $\nu = 0.6$ THz; here, $\textbf{r}$ is a radius vector in the waveguide cross-section plane. The measured intensity pattern is limited by the input aperture and smaller than its simulated counterpart. Nevertheless, it is obvious that the significant part of the incident beam is coupled to the fundamental guided-mode in our experiments, because for our waveguiding configuration the power coupling coefficient of LP$_{02}$ higher-order modes is almost 10 times lower than coupling coefficient of the fundamental mode.

For the metal-coated waveguide, in Fig. 4d, the measured propagation loss $\alpha \left( \nu \right)$ show high-to-moderate agreement with our numerical predictions. In fact, quite good agreement between the experimental and theoretical loss is observed in the $\nu =0.57$–0.65 THz range (excluding the frequencies near the 0.62 THz) with the minimal experimental value of $5.0 \pm 1.0~$dB/m at 0.6 THz. At higher frequencies, significant discrepancies between the experiment and theory are notable, mainly due to the low signal-to-noise ratio of the used BWO at the frequencies higher then 0.67 THz. However, the metal-sapphire waveguide still demonstrates lower sensitivity to the fabrication-induced variations in its geometry at distinct frequencies. Panels (e) and (f) show the experimental and theoretical mode intensities $I \left( \textbf{r} \right)$ at 0.6 THz as well. Some asymmetry in the experimental intensity pattern could be attributed to the inhomogeneities of the input THz beam and its spatial misalignment towards the studied waveguide. Nevertheless, the observed discrepancies are quite small.

Additional sharp resonance peaks in the experimental $\alpha \left( \nu \right)$-curves at higher frequencies above 0.68 THz with a smaller period might be associated with the longitudinal standing waves in the finite-length waveguide fragment. In fact, their resonant frequencies can be defined as $m c_{0} / \left( 2 L n_\textrm{eff} \right)$; here, *m* is a positive integer, $c_0 = 3 \times 10^8$ m/sec is the speed of light in free space, $L \sim 1$–10 cm is the waveguide length, and $n_\textrm{eff}$ is the effective refractive index of a guided mode, which is commonly close to unity ($n_\textrm{eff} \simeq 1$) for ARROW or photonic crystal waveguides with a mode confined in a hollow core. These resonances are caused by the applied double-pass scheme of spectroscopy (Fig. 3a). They are poorly resolved in our experimental data, differ considerably from the resonances of the ARROW mechanism, and might be the reason of some experimental overestimation of propagation loss $\alpha$. Another problem is beam defocusing at frequencies higher than 0.68 THz. In this case, the significant angular beam distortion leads to higher-order mode generation in the waveguide, non-equivalence of the coupling/de-coupling conditions for the two waveguide samples, and, as a result, to distortions in the loss calculation results.

## Discussions

**Figure 5:**
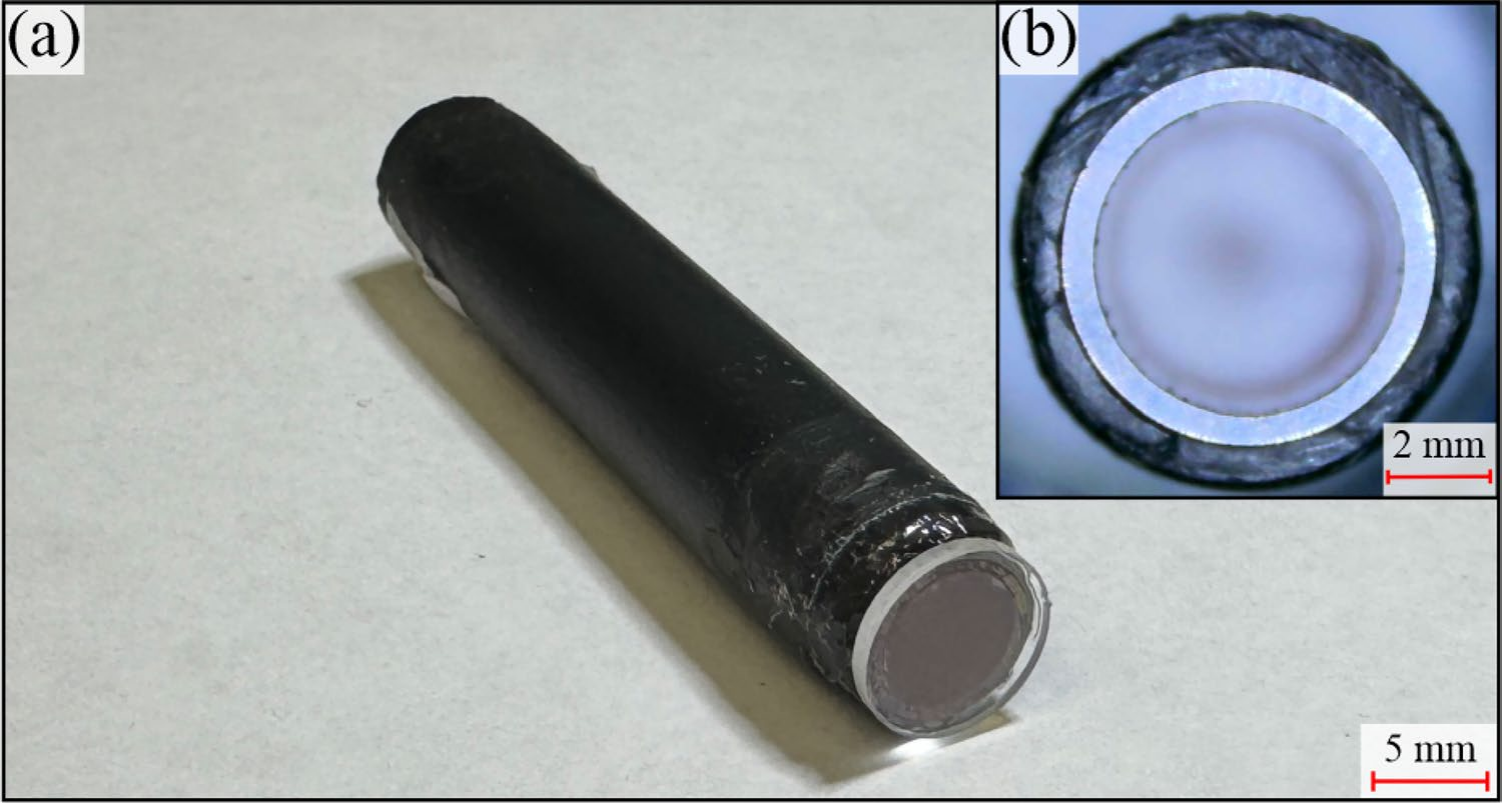
50 mm long THz endoscope made of the ARROW THz waveguide exploiting the described metal-coated geometry with the probe sapphire window glued to its end. (**a**) Photo of the endoscope. (**b**) Microscopy of the waveguide cross-section.

Thereby, both the experimental and numerical data on the optical performance of the developed waveguides demonstrate their potential in different THz applications, such as the THz endoscopy and Fabry-Pérot refractometry^4,22^. Among them, the metal-coated arrangement is preferable due to slightly smaller loss and insensitivity of the fundamental guided mode to external obstacles and environments.

Despite the ARROW or photonic-crystal waveguides with multi-period cladding also demonstrate a low loss (namely, at a few dB/m level or slightly less), they remain inconvenient for remount sensing^63–66^. The deposition of an outer metal coating offers a simple straightforward approach to enhance the guiding properties and, thus, meet the demands posed by the THz endoscopy and remote sensing^42,67^. Furthermore the propagation loss remains on the low level, once the antiresonance condition is satisfied. However, rigidity of the considered waveguide designs forms their major drawback significantly limiting their utility.

This work demonstrates that all fabrication-induced deviations in the cross-section geometry significantly impact the waveguide performance. On the one side, accurate post-treatment of the cross-section elements is required to ensure the desired performance, but the difficulties in sapphire treatment could limit the applicability and cost-efficiency of the resultant waveguides. In fact, this effect is general and should be inherent to the ARROW waveguides based on the different polymer and crystalline materials and fabrication strategies. On the other side, similarly to the disorder photonic crystal waveguides with broaden bandgaps^68^, one can design the ARROW waveguide with slightly disordered cladding layers (i.e., variable thicknesses of these layers, over the waveguide length, or upon rotation towards the optical axis) to suppress the resonant loss peak and ensure broader operation, while consciously allowing for the increased loss average loss. Such an opportunity forms a topic of the separate research. The waveguides and fibers designed for the THz endoscopic application ^4,22^ should meet the following key requirements:

- Ability to transmit THz radiation with low loss ($\le 1-10$ dB/m)) over considerable (up to 1 m) distances. Furthermore, to transmit broadband THz pulses low dispersion ($\le 1$ ps(THz cm$^{-1}$) is in order. These properties enable the delivery of THz radiation to the hard-to-access tissues and targeted internal placed in contact with the distal end of the endoscope, as well as the detection of the reflected THz signal with an adequate signal-to-noise ratio.
- Small outer diameter ($\le 3-6$ mm) and (preferably) flexibility of the waveguide/fiber. This enables their integration into minimally invasive endoscopy and (in the case of rigid waveguides) laparoscopy. Bending and shrinkage losses should be minimal and have a negligible effect on the guiding properties of the fiber. In fact, the development of flexible THz endoscopes with adequate optical performance seems to be a very daunting test, but even hard THz endoscopes can meet the demands of many real-world THz applications.
- Absence of radiation coupling to the external environment. Such coupling alters the modal characteristics due to interactions with various external objects and environments. Indeed, variation of the guiding characteristics of a waveguide due to interactions with external obstacle during measurements hampers the THz measurements and discrimination between healthy and pathological tissues, with the typically small ($\sim 1-5~\%$) contrast between them.
- Chemical inertness to biological tissues and liquids, and resistance to medical sterilization conditions. The proposed instruments should withstand repeated exposure to temperatures of up to $190~ ^{\circ }$C, the pressure up to 2 Bar, and the chemicals like ethylene oxide, or alternative sterilization methods ^69^.

To enable THz measurements in aggressive environments, an additional protective coating may be in order. A thin metal layer on the waveguide surface can effectively mitigate the potential influence of this protective layer on the optical properties. As an example, Fig. 5a shows a THz Fabry-Perot endoscope with the lengths of 50 mm and a sapphire window glued at the output end^22^. This endoscope is made of the metal-coated waveguide covered by the chemically stable polymer shrink and hermetic glue layer. From panel (b), one notice that the shrink has a non-uniform thickness, but the the optical properties of the endoscope depend solely on the dimensions of the sapphire tube. Detailed analysis of this endoscope is postponed to our future work.

An important problem to be addressed in our future studies is the long-term environmental stability of the developed waveguides in the context of different THz applications. This stability should be examined experimentally considering different harsh environments, such as biological tissues and liquid in the THz medical diagnosis. Approaches to improve the stability should also be developed, such as the formation of protective polymer coatings or oxide films on the metal surface of the waveguide. Nevertheless, such developments form the topic of separate full-blown work.

Finally, we notice that a favorable combination of the low propagation loss and the small outer diameter of the developed ARROW THz waveguides makes them promising candidates for a variety of applications, such as the endoscopic measurements and exposure of hard-to-access tissues and internal organs^4,11,13,22,70^.

## Conclusions

In conclusion, using the EFG-grown hollow-core sapphire tube, as a key element, we developed the two distinct hollow-core ARROW THz waveguides – namely, the all-dielectric and metal-coated ones formed by coating the tube by the PTFE- and copper layers, respectively. These coatings aim at increasing the waveguidance efficiency and preventing the guided-mode leakage into the surrounding environment, as well as underlie the distinct optical performance of the two waveguides. Moreover, the outer metal coating completely prevents mode leakage, which forms advantage of the metal-coated arrangement over the all-dielectric one, in which the evanescent field extends to the outer space and, thus, can lead to the guided-mode de-coupling due to its interactions with any external obstacle. Both waveguides are studied numerically and experimentally in the 0.56–0.70 THz range. They operate in effectively single-mode regime. The discrepancies between the theoretical and experimental data are attributed to fluctuation of the cross-section geometry over the waveguide length caused by manufacturing inaccuracies. The developed ARROW THz waveguides hold a potential in THz remote sensing and exposure of hard-to-reach objects.

## Supplementary Information

Supplementary Information 1.

## Data Availability

The data that support the findings of this study are available from the corresponding author upon reasonable request.
