# Supplementary material for: Hollow-core antiresonant THz waveguides based on polymer- and metal-coated sapphire tube
Source: Sci Rep. 2026 Apr 27;16:19399. doi: 10.1038/s41598-026-50305-8 (PMC13287482; doi:10.1038/s41598-026-50305-8)
Supplement: Supplementary file 1 — Supplementary Information 1. [file 41598_2026_50305_MOESM1_ESM.docx]

SUPPLEMENTARU MATERIAL

**Hollow-core antiresonant THz waveguides based on polymer- and metal--coated sapphire tube**

Gleb M. Katyba^1,*^, Anna S. Kucheryavenko^1,2^, Alexander N. Golikov^1^, Anna

N. Rossolenko^1^, Vladimir N. Shilov^1^, Kirill B. Dolganov^2^, Maria G. Burdanova^3^, Igor

E. Spektor^2^, Vladimir N. Kurlov^1^, and Kirill I. Zaytsev^2^

*^1)^Osipyan Institute of Solid State Physics of the Russian Academy of Sciences, Russia*

*^2)^Prokhorov General Physics Institute of the Russian Academy of Sciences, Russia*

*^3)^* *Moscow Center for Advanced Studies, 123592, Moscow, Russia*

*^a)^E-mail:* [*katyba_gm@issp.ac.ru*](mailto:katyba_gm@issp.ac.ru)

In this document, we provide details of the sapphire shaped crystals fabrication via the edge-define film-fed growth (EFG) technique. Namely, you could find photo of the polished sapphire tubes.

| 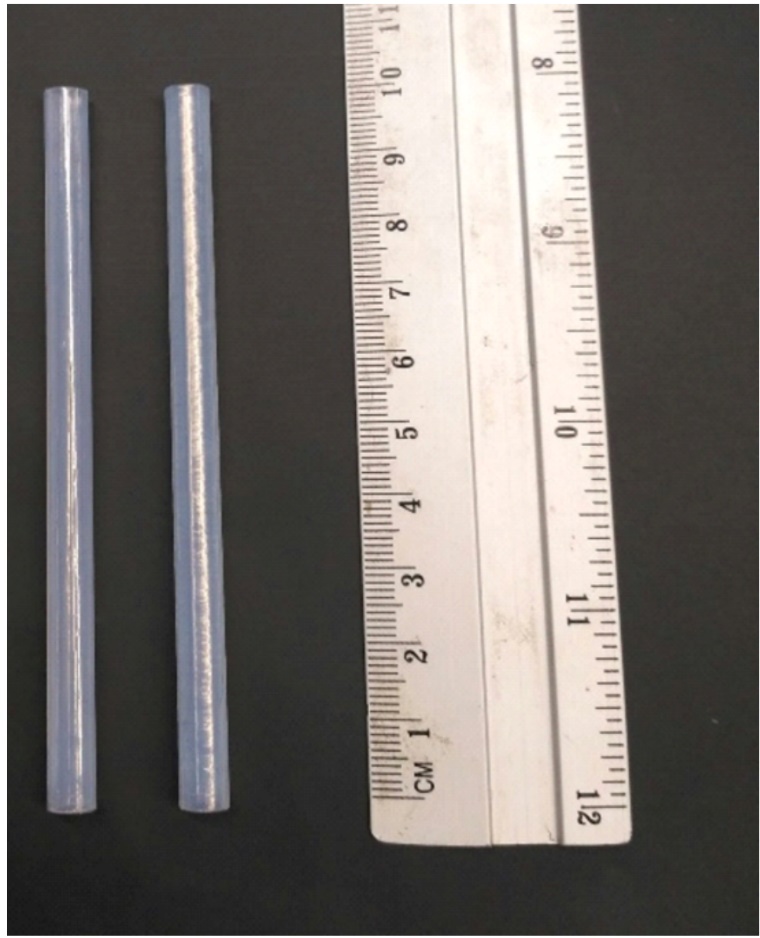 |
| --- |
| Figure 1S. Photo of the two polished sapphire tubes which used as a basis for THz waveguides. |
